# Effect of Nanoconfinement of Polyphenolic Extract from Grape Pomace into Functionalized Mesoporous Silica on Its Biocompatibility and Radical Scavenging Activity

**DOI:** 10.3390/antiox9080696

**Published:** 2020-08-03

**Authors:** Ana-Maria Brezoiu, Laura Bajenaru, Daniela Berger, Raul-Augustin Mitran, Mihaela Deaconu, Daniel Lincu, Anicuta Stoica Guzun, Cristian Matei, Mihaela Georgeta Moisescu, Ticuta Negreanu-Pirjol

**Affiliations:** 1 Department of Inorganic Chemistry, Physical-Chemistry & Electrochemistry, Faculty of Applied Chemistry and Materials Science, University “Politehnica” of Bucharest, 1-7 Gheorghe Polizu Street, 011061 Bucharest, Romania; anamariabrezoiu@gmail.com (A.-M.B.); georgianabarbulescu@gmail.com (L.B.); mihaela_deaconu@yahoo.com (M.D.); daniel.lincu1113a@gmail.com (D.L.); cristian.matei@upb.ro (C.M.); 2 “Ilie Murgulescu” Institute of Physical Chemistry, Romanian Academy, 202 Splaiul Independentei Street, 060021 Bucharest, Romania; raul.mitran@gmail.com; 3 Department of Chemical and Biochemical Engineering, Faculty of Applied Chemistry and Materials Science, University “Politehnica” of Bucharest, 1-7 Gheorghe Polizu Street, 011061 Bucharest, Romania; anicuta_s@yahoo.com; 4 Department of Biophysics and Cellular Biotechnology, “Carol Davila” University of Medicine and Pharmacy, 8 Eroii Sanitari Blvd., 050474 Bucharest, Romania; mihaela.moisescu@umfcd.ro; 5 Biophysics and Cellular Biotechnology Research Excellence Center, “Carol Davila” University of Medicine and Pharmacy, 8 Eroii Sanitari Blvd., 050474 Bucharest, Romania; 6 Faculty of Pharmacy, “Ovidius” University of Constanta, Aleea Universitatii No. 1, 900470 Constanta, Romania; ticuta_np@yahoo.com

**Keywords:** polyphenolic extract, functionalized mesoporous silica, radical scavenger activity, polyphenolic extract encapsulation, polyphenols delivery profile

## Abstract

The aim of this paper is to assess the properties of Mamaia (MM) grape pomace polyphenolic extract loaded onto pristine and functionalized MCM-41 mesoporous silica as potential ingredients for nutraceuticals or cosmetics. The chemical profile of hydroalcoholic polyphenolic extracts, prepared either by conventional extraction or microwave-assisted method, was analyzed by reverse-phase high-performance liquid chromatography with photodiode array detector (HPLC-PDA) analysis, while their radical scavenger activity (RSA) was evaluated using DPPH (2,2-diphenyl-1-picrylhydrazyl radical) and ABTS (2,2′-azino-bis(3-ethylbenzothiazoline-6-sulphonic acid) assays. The extract-loaded materials were characterized by Fourier transform infrared (FTIR) spectroscopy, N_2_ adsorption-desorption isotherms, thermogravimetric analysis, as well as RSA (DPPH and ABTS assays). The polyphenols release profiles from pristine and functionalized (with mercaptopropyl, propyl sulfonic acid, cyanoethyl and propionic acid moieties) MCM-41-type supports were determined in phosphate buffer solution (PBS) pH 5.7. For selected materials containing embedded phytochemicals, cellular viability, and oxidative stress level on immortalized mouse embryonic fibroblast cell line (NIH3T3) were evaluated. A more acidic functional groups linked on silica pore walls determined a higher amount of phytochemicals released in PBS. The extract-loaded materials showed a good cytocompatibility on tested concentrations. The embedded extract preserved better the RSA over time than the free extract. The polyphenols-loaded MCM-41-type silica materials, especially MM@MCM-COOH material, demonstrated a good in vitro antioxidant effect on NIH3T3 cells, being potential candidates for nutraceutical or cosmetic formulations.

## 1. Introduction

During the winemaking process, sugars from grapes are converted in alcohol, but the main bioactive compounds are not greatly affected by the fermentation, almost 70% of phytochemicals being preserved [1,2,3]. Grape pomace, a mixture of skins, pulp, seeds, and stems, obtained after pressing of grapes in the winemaking process, represents about 20–30% wt of processed grapes [1,4]. The valorization of grape pomace is important considering either the environmental issues [5] or the applications of its bioactive compounds [6,7,8]. However, in the last decade only 3% of grape pomace was turned into animal feed [9,10], as compost [11,12,13], or other applications as thermal insulation for buildings [14]. Grape pomace is an abundant and valuable source of polyphenolic compounds, which are receiving an increasing interest due to their health benefits exerting antioxidant, anti-inflammatory, antibacterial, anticarcinogenic, antidiabetic, and cardioprotective effects [15,16,17,18,19], and great potential for food and cosmetics industries.

Phenolic compounds are secondary plant metabolites and can be used as adjuvants in various diseases like diabetes, brain aging diseases or hypertension. Polyphenols have a chemo-preventive effect on cancer progression, maintaining healthy cells and preventing the tumor growth, angiogenesis and metastasis [15]. For instance, gallic acid has shown significant anticancer activity in leukemia [16] and prostate cancer [17], and recently, was tested in vitro in a gel formulation against skin melanoma [18]. *p*-Coumaric acid was evaluated against colon cancer and proved to retard the cell cycle progression [19], while quercetin was tested against pyruvate dehydrogenase kinase 3, an enzyme involved in cancer cell progression demonstrating a viability decrease of human hepatic cancer cells and adenocarcinomas [20]. Also, quercetin-loaded chitosan nanoparticles showed enhanced cytotoxicity in comparison with the quercetin alone against human lung and breast cancer cell lines, shrinking the tumor size in mice lung and tumors from breast xenograft [21].

Inflammation is a natural response of the organism at external stimuli like irritations, infections, or injuries, which leads to a proinflammatory cytokines release. The overproduction of these cytokines usually determines severe disorders like allergies, arthritis, or atherosclerosis etc. Plant phytochemicals can be used to inhibit the cytokines overproduction, having an anti-inflammatory effect [15]. Thus, an in vivo study showed that *p*-coumaric acid was efficient against the expression of tumor necrosis factor (TNF-α) involved in arthritis [22]. A similar anti-inflammatory effect was reported for caffeic and ellagic acids leading to a decrease of interleukins and TNF-α activities, when applied in mice diets [23]. Furthermore, rutin and hesperidin showed an anti-inflammatory effect when they used to treat *cis*-platin induced nephrotoxicity in rats [24].

Usually, antibacterial, or antiviral agents either kill or slow down the bacteria or virus multiplication without damaging any surrounding cell or tissues. Methyl gallate proved a good activity against herpes viruses [25], or in association with ciprofloxacin exhibited a strong antimicrobial potential against several *Salmonella* strains [26]. Also, polyphenolic compounds from flavonoids group demonstrated efficiency on the inhibition of very well-known bacteria, *Salmonella*, *Clostridium* or *Bacillus* [27].

Several polyphenolic substances were tested in the treatment of diabetes. For instance, caffeic and ellagic acids were used against kidney disease caused by diabetes at rats showing efficiency after 12 weeks’ treatment [23]. Resveratrol exhibited anti-diabetes potential at rats, modulating the gene involved in the blood homeostasis [28]. Other polyphenols, like (+)catechin, (−)epicatechin, chlorogenic acid, isoflavones, and tannic acid were involved in the reduction of the glucose transport using sodium-coupled glucose transporter (SGLT1) [29]. Catechins from green tea proved a strong neuroprotective effect and could prevent Parkinson’s and Alzheimer’s diseases [30,31]. Similar effects were reported for a grape leaf extract in AlCl_3_ induced Alzheimer’s disease [32]. Quercetin could be used in hypertension treatment considering its activity on angiotensin-converting enzyme [33], having also a cardioprotective effect through attenuation of the metalloproteinase-1 expression [34].

The antioxidant activity is one of the most notable features of polyphenolic extracts that act through several mechanisms: free radical scavenging, inhibition of lipid peroxidation, and lowering of hydroperoxide formation [35]. Polyphenols act against lipid peroxidation through a complex mechanism in which they (catechins, epicatechins and procyanidin B dimer) and lipid bilayer form rigid structures [36]. Self-preservation characteristics of polyphenolic extracts recommend them as ingredients in food, cosmetic or pharmaceutical formulations [37].

In the last years, various formulations based on grape pomace extracts for food products were proposed. For example, a grape pomace extract encapsulated in nanoemulsions based on soy protein and chitosan was successfully used in commercial fruit juices with an enhanced antioxidant activity [38]. The utilization of grape pomace flour or aqueous extract as functional ingredients in yoghurt showed a good acceptance from the tested subject group, emphasizing that the extract exhibited an enhanced bioavailability based on simulated digestion tests [7]. Also, a polyphenolic extract encapsulated in mixtures of arabic gum and maltodextrin demonstrated better stability after 75 days of storage in moist conditions [39].

In cosmetics, grape-derived products are added in low concentration, up to 3% [40]. Maluf et al. investigated the efficiency and safety of the use of grape pomace extracts in cosmetic formulations, and concluded that the extract prepared in 75% acetone-water mixture exhibited an antioxidant activity better than a common antioxidant, butylated hydroxyanisole, used in cosmetics, and a cytoprotective effect on 3T3 cells line [41]. Using a 70% hydroethanolic extract from grape pomace combined with other filters for sunscreen formulations led to the obtaining of a sun protecting factor (SPF) of 76, 2.5 times higher than the formulation without adding grape pomace extract. However, without adding additional sun filters, no SPF protection was achieved [42].

Among matrices employed for encapsulation of biomolecules (enzymes, peptides, genes, vitamins, etc.), mesoporous silica nanoparticles (MSN) were widely studied to design versatile platforms for various medical applications [43,44]. Tailoring the MSN surface properties by functionalization with organic groups/molecules [45,46] is a major advantage to achieve appropriate biomolecules–carrier interactions, sensitivity to stimuli produced by biological systems, which could facilitate the delivery of cargo molecules in a targeted tissue, leading to an enhanced therapeutic efficiency [47].

Recently, we reported the enhancement of thermal stability along with the preservation of radical scavenging activity for polyphenolic extracts by embedding them into mesopores of silica modified with Zn, Mg or Ce heteroatoms [48]. Here, we report for the first time, the loading of a hydroalcoholic polyphenolic extract from grape pomace into mesopores of MCM-41-type silica functionalized with organic groups and how the acidic silica surface properties influence the release of polyphenolic compounds from carrier in phosphate buffer solution. We demonstrated a good cytocompatibility (MTS assay, where MTS is an abbreviation for 3-(4,5–dimethylthiazol–2-yl)–5-(3-carboxymethoxyphenyl)–2-(4-sulfophenyl)-2H-tetrazolium) and in vitro antioxidant activity on NIH3T3 cells line of materials containing embedded polyphenolic extract that could be further used for the development of nutraceutical and cosmetic formulations.

## 2. Materials and Methods

### 2.1. Materials

For the synthesis of mesoporous silica, tetraethyl orthosilicate (TEOS, Fluka, Seelzer, Germany), trimethylhexadecylammonium bromide (CTAB, Alfa Aesar, Ward Hill, MA, USA), NH_4_Cl, 36.5–38 % wt hydrochloric acid (Merck Group, Darmstadt, Germany), and 25% wt ammonia aqueous solution (Scharlau, Scharlab S.L., Barcelona, Spain) were used as received without further purification. For mesoporous silica functionalization, MCM-41 (Merck Group, Darmstadt, Germany) and (3-mercaptopropyl) triethoxysilane (MPTES, 95%), (3-cyanoethyl) triethoxysilane (CETES, 97%), toluene, sulfuric acid 98% were purchased from Sigma-Aldrich (Sigma-Aldrich Co. Merck Group, Darmstadt, Germany) and hydrogen peroxide 30% from Fluka (Seelzer, Germany). For extract recovery experiments potassium phosphate diacid and sodium phosphate monoacid (Sigma-Aldrich Co. Merck Group, Darmstadt, Germany) were used.

The reagents used in the spectrophotometric determinations were sodium carbonate (Na_2_CO_3_), potassium persulphate (K_2_S_2_O_8_), Folin–Ciocalteu reagent, 2,2-diphenyl-1-picrylhydrazyl (DPPH), and 2,2′-azino-bis (3 ethylbenzothiazoline-6-sulphonic acid) (ABTS) purchased from Sigma-Aldrich (Sigma-Aldrich Co. Merck Group, Darmstadt, Germany) and 6-hydroxy- 2,5,7,8-tetramethylchroman-2-carboxylic acid (Trolox, 97%, Aldrich Chemical Co Inc., Milwaukee, WI, USA). Ascorbic acid powder was bought from a local vendor.

For chromatographic analyses, several standard high-performance liquid chromatography (HPLC)-grade compounds were used: Phenolic acids: gallic acid (98%, Alfa Aesar, Ward Hill, MA, USA), protocatechuic acid (>98%, HPLC, TCI, Tokyo, Japan), caftaric acid (Molekula GmbH, Munich, Germany), vanillic acid (>98%, GC, TCI, Tokyo, Japan), syringic acid (>98.5%, Molekula GmbH, Munich, Germany), hydroxycinnamic acids: caffeic acid (98%, HPLC, Sigma, Merck Group, Darmstadt, Germany), *trans-p*-coumaric acid (analytical standard, Sigma-Aldrich Co. Merck Group, Darmstadt, Germany), *trans*-ferulic acid (>98%, GC) and chicoric acid (>98%) from TCI (Tokyo, Japan), chlorogenic acid (primary reference standard, HWI group, Alpen Aan de Rijn, The Netherlands), rosmarinic acid (>98%, HPLC, Sigma, Merck Group, Darmstadt, Germany), flavonoids: catechin hydrate (>98%, HPLC, Sigma, Merck Group, Darmstadt, Germany), (−) epicatechin (>98%, HPLC, TCI, Tokyo, Japan), quercetin (>95%, HPLC), rutin hydrate (95%, HPLC), myricetin (>96%, HPLC-grade), kaempferol (>97%, HPLC) from Sigma (Merck Group, Darmstadt, Germany), tannins: ellagic acid dihydrate (>98%, HPLC, TCI, Tokyo, Japan), stilbenoids: *trans*-resveratrol (certified reference material, Sigma-Aldrich Co. Merck Group, Darmstadt, Germany), anthocyanidins: cyanidin chloride (>95%, HPLC, Sigma, Merck Group, Darmstadt, Germany), malvidin chloride (>95%, HPLC, Sigma-Aldrich Co. Merck Group, Darmstadt, Germany), pelargonidin chloride (Aldrich Chemical Co Inc., Milwaukee, WI, USA) and delphinidin chloride (analytical standard, Sigma-Aldrich Co. Merck Group, Darmstadt, Germany) and solvents like ethanol, acetonitrile (ACN) were purchased from (Riedel-de Haën, Honeywell Riedel-de Haën, Seelzer, Germany), and formic acid (Merck Group, Darmstadt, Germany) were used without additional purification. For all solutions and experiments, ultrapure water (Millipore Direct- Q3 ultraviolet (UV) water purification system with Biopack UF cartridge) was used.

The grape pomace from Mamaia cultivar, the residue resulted from the production of a rosé wine, was kindly offered by Research Station for Viticulture and Oenology Murflatlar (Black Sea region, Romania).

### 2.2. Preparation and Characterization of Polyphenolic Extracts from Mamaia Grape Pomace

The hydroalcoholic polyphenolic extracts were prepared using a grape pomace from Mamaia cultivar, MM, (Black Sea region, 2017) by both conventional (Conv) and microwave-assisted (MW) extraction. The conventional extraction was performed by mixing the vegetal material with water–ethanol 1/4 *v*/*v* at room temperature overnight, followed by a reflux heating in three stages of 1h (vegetal material/ethanol overall ratio of 1/18 *w*/*v*), under constant magnetic stirring, intermediate filtration and solvent replacement in the same amount and then extract fractions were stored separately. Another extraction with all fractions combined was carried out and the extract was labelled MM(Conv). The MW extraction was done in a microwave reactor (Milestone Technologies Neos Essential Oil Systems, Shelton, CT, USA) using the grape marc and ethanol-water mixture in the same ratio as for conventional extraction, in three stages of 15 min. using a MW power of 75 W with intermediate filtration, solvent replacement and then the fractions were stored together, the extract being denoted MM(MW). The polyphenolic extracts or fractions were dried under vacuum until a constant mass, and then re-dissolved in ethanol-water 4/1 *v*/*v*.

The polyphenolic extracts were characterized by several spectrophotometric methods (Shimadzu UV-1800, Shimadzu Corporation, Kyoto, Japan) to determine the total polyphenols, total reducing substances, flavonoids, as well as anthocyanins content, while the extract composition was assessed using reversed-phase high-performance liquid chromatography with a photodiode array detector (HPLC-PDA, Shimadzu Nexera 2, Shimadzu Corporation). The description of the spectrophotometric methods and HPLC analysis were previously reported in our paper [49] and the chromatographic method was further improved with the addition of four new standard substances.

### 2.3. Synthesis of Pristine and Functionalized MCM-41 Supports

Pristine MCM-41 silica was synthesized by the sol-gel method using tetraethyl orthosilicate and hexadecyltrimethylammonium bromide as silicon precursor and structure directing agent, respectively, according to a described procedure [50]. The template agent was removed by extraction in NH_4_Cl saturated ethanolic solution (2 h at reflux and 1 h in an ultrasonic bath at 60 °C), followed by a second extraction step assisted by ultrasounds (in an ultrasonic bath) in 4% HCl ethanolic solution at 60 °C. The resulted silica material was denoted MCM-41E.

The mercaptopropyl-functionalized silica material, MCM-SH, was prepared by post-synthesis method using commercial MCM-41 and (3-mercaptopropyl) triethoxysilane (MPTES) in toluene at reflux, 4 h, using toluene:silica:organosilane molar ratio of 230:5:1. MCM-SO_3_H material was obtained from MCM-SH sample through oxidation of thiol groups to sulfonic moieties, at room temperature, using H_2_O_2_ acidic aqueous solution [51].

The cyanoethyl functionalized silica material, MCM-CN, was obtained in the same conditions as MCM-SH in toluene at reflux for 16 h, using (3-cyanoethyl) triethoxysilane (CETES) as functionalization agent, while the support functionalized with carboxyl groups, MCM-COOH, was prepared from an MCM-CN sample in H_2_SO_4_ 50% aqueous solution (1 g MCM-CN in 30 mL H_2_SO_4_ 50%) at reflux for 16 h by a method that we recently reported [52].

### 2.4. Loading Polyphenolic Extracts into Mesoporous Silica-Type Nanocarriers

As supports for loading of polyphenolic extracts, pristine MCM-41E silica and mesoporous materials functionalized with propionitrile groups, MCM-CN, propionic acid moieties, MCM-COOH, mercaptopropyl groups, MCM-SH, and propyl sulfonic acid moieties, MCM-SO_3_H, were used.

The materials containing polyphenolic extract were prepared by an incipient wetness impregnation method using MM(Conv) extract. Briefly, the polyphenolic extract having a concentration of 13 mg/mL was mixed with different silica matrices, previously outgassed at 110 °C for 16 h, homogenized with a stirring rod, and the resulting suspension was dried under vacuum for 10 h, in static conditions. The materials containing embedded polyphenolic extract resulted after vacuum drying were denoted MM@support.

### 2.5. Materials Characterization

The mesoporous carriers were characterized using small-angle X-ray diffraction (XRD), Fourier transform infrared (FTIR) spectroscopy, nitrogen adsorption–desorption isotherms, scanning electron microscopy (SEM), and thermogravimetric analysis (TG), whereas the extract-loaded materials were investigated by FTIR spectroscopy, N_2_ adsorption-desorption isotherms, TG and radical scavenger activity determination (DPPH and ABTS assays).

Small-angle XRD analyses were performed on a Rigaku MiniFlex II diffractometer (Rigaku Corporation, Tokyo, Japan) with Cu-Kα radiation in the range of 1.2–6.0° (2*θ*), using a scanning rate of 0.5°/min and a 0.01°/step. The FTIR spectra were recorded in 4000–400 cm^−1^ range on a Bruker Tensor 27 spectrophotometer (Bruker Corporation Optik GmbH, Bremen, Germany, KBr pellet technique). Nitrogen adsorption–desorption isotherms were recorded at 77 K using a Quantachrome Autosorb iQ_2_ gas sorption analyzer (Quantachrome Instruments, Boynton Beach, FL, USA). The specific surface area values, S_BET_, were computed through the Brunauer Emmett Teller method in the relative pressure range of 0.05–0.25, while the pore size distribution curves and the average pore diameter, *d*_DFT,_ were determined by non-local density functional theory (NLDFT) from the isotherm adsorption branch and the total pore volume was measured at *p*/*p*_0_ = 0.99. Prior to recording the isotherms, the mesoporous supports and extract-loaded samples were outgassed at 110 °C, 12 h and 35 °C, 17 h, respectively.

The morphology of carriers was investigated by SEM on a Tescan Vega 3 LMH microscope (Brno, Czech Republic) with an EDX detector for the determination of chemical composition.

The supports, extract-loaded samples and materials remained after extract recovery experiments were also characterized by thermogravimetric analysis performed on Mettler Toledo GA/SDTA851e (Greifensee, Switzerland) equipment in the temperature range of 25–600 °C in air, at a heating rate of 10 °C/min.

The FTIR spectra of materials containing embedded extract were used to evidence the organic molecules adsorption from the polyphenolic extract onto the supports. Thermogravimetric analyses (TG) were carried out to determine the amount of polyphenolic compounds embedded into mesoporous silica matrices and the nitrogen adsorption-desorption isotherms were recorded to check the filling of mesopores with natural compounds from polyphenolic extracts.

The radical scavenger activity (RSA) of extract-loaded materials was evaluated by DPPH and ABTS methods applied for solid samples. The detailed procedure for RSA determination by DPPH assay is presented elsewhere [49]. In brief, the extract-loaded material was analyzed in comparison with the corresponding support and the free extract in the same amount as in the material containing extract, using as control the degradation of DPPH free radical solution, after 24 h. The experiments were carried out in closed containers, under stirring on an orbital shaker, in dark conditions. Afterwards, aliquots of all types of samples were withdrawn, centrifuged for 15 min, and then the solution absorbance was measured at 517 nm.

The more reliable radical scavenger activity of samples is better evaluated by various methods. Hence, the ABTS assay was also adapted for solid samples. As in the case of ABTS method applied for free extracts, ABTS carbocation radical was generated using 176 µL of 2.45 mM potassium persulfate (K_2_S_2_O_8_) added at 10 mL 7 mM aqueous ABTS solution and the reaction mixture was kept under constant magnetic stirring, in the dark conditions, for 20 h prior its use. Then, the corresponding mass of the extract-loaded material (containing the same extract amount as in 50 μL extract of chosen concentration for 2.95 mL ABTS free carbocation radical solution) was considered. The same amount of silica-type support as in the extract-loaded material was tested in the same conditions to assess its influence on the RSA. After the addition of aqueous ABTS^•^^+^ solution (diluted accordingly to have an absorbance of 0.7) on the solid samples or free extract, the experiments were carried out in closed containers, in dark conditions, and kept for 6 h on an orbital shaker, using ABTS^•^^+^ solution as control. Afterwards, aliquots of samples were withdrawn, centrifuged for 15 min and the solution absorbance was measured at 730 nm. Statistical analysis of the data was performed using Student’s *t*-test on each pair of interest. Differences were considered statistically significant for which *p* < 0.05.

### 2.6. Determination of Polyphenols Release Profiles

The release fluid, phosphate buffer solution pH 5.7, was chosen considering the interest of polyphenols delivery from silica-type carriers in small intestine as adjuvant for cancer therapy, close to tumors, pH being more acidic because of a higher rate of glycolysis than in healthy tissues [53] or for the inclusion of polyphenol-loaded nanoparticles in cosmetics or nutraceuticals.

The release experiments of polyphenols from pristine and functionalized silica were performed in 0.2 M saline phosphate buffer solution (PBS) pH 5.7, under constant magnetic stirring (200 rpm), at 37 °C, in dark conditions. Each material containing extract (equivalent amount of 10 mg extract) was immersed in 20 mL PBS. At set time intervals, samples of 100 μL of release medium were withdrawn (substituted by 100 μL buffer solution) and properly diluted (up to 1 mL) with PBS pH 5.7, and then analyzed by ultraviolet–visible (UV-VIS) spectroscopy at 280 nm corresponding to a total polyphenolic index determination [54]. The amount of extract recovered at any time was determined based on a calibration curve of MM(Conv) extract in buffer pH 5.7 (*y* = 0.0053 × *x*, *R*^2^ = 0.9990) for a concentration of extract in the range of 10–300 µg/mL, at 280 nm wavelength.

### 2.7. Evaluation of Cellular Viability and Oxidative Stress Level

The NIH3T3 cells (NIH 3T3 4-2, ECACC 86041101, Salibury, UK) were grown for 24 h to half-confluence in 12-well plates (665180, Greiner Cellstar, Merck Group, Darmstadt, Germany) using Dulbecco’s Modified Eagle Medium high glucose, with l-glutamine (D5796, Sigma, Merck Group, Darmstadt, Germany) (DMEM) supplemented with 10% fetal bovine serum (D7524, Sigma, Merck Group). Growing medium was replaced with DMEM containing either suspension of extract-loaded materials, mesoporous supports, or polyphenolic extract. The day of experiments, stock solutions of extract-loaded materials and mesoporous supports were prepared in 1 mL DMEM and homogenized (45 min, room temperature) using an ultrasonic bath (621.05.001, Isolab, Eschau, Bavaria, Germany). Stock solution of hydroalcoholic polyphenolic extract was kept in the dark, at 4 °C. Two concentrations of polyphenolic extract in culture medium, 100 µg/mL and 50 µg/mL, were used. The concentrations of extract-loaded materials were calculated to correspond to an amount of embedded extract similar to that used for polyphenols extract. The concentrations of mesoporous carriers were calculated to correspond to the amounts of the support added to the embedded extract samples. Two controls were used: cells grown in DMEM without any compound, and cells grown in DMEM with alcohol in concentration corresponding to the highest concentration of the alcohol from the extracts. After the 24 h incubation period, the wells were washed twice with 0.9% NaCl solution and the cells were incubated for 2 h at 37 °C with a mix obtained by diluting the CellTiter 96^®^Aqueous One Solution (Promega Corporation, Madison, WI, USA) in a proportion of 1/5 with culture medium. The concentration of soluble formazan produced by viable cells was quantified by measuring the absorbance at 492 nm using a plate reader (Stat Fax 3200, Awareness Technology Inc., Palm City, FL, USA). Before measurements, the cell medium with formazan was transferred into 96-well plates (TPP, Merck Group, Darmstadt, Germany). After subtracting the blank value, the absorbances were normalized by dividing the value of each well by the average absorbance of the controls (*w/o* any compound).

To evaluate the intracellular reactive oxygen species (ROS) production, the same procedure of cell seeding and exposure to compounds as for cell viability assay was applied. After 24 h of incubation and washing, the cells were incubated for 20 min, at 37 °C, with a solution of 100 µM 2′,7′-dichlorofluorescin diacetate (H_2_DCFDA, Sigma-Aldrich Co. Merck Group, Darmstadt, Germany) in medium without phenol red (DMEM *w/o* phenol red, 21063-029, Gibco, Thermo Fisher Scientific Inc., Waltham, MA, USA). Then, the cells were gently washed, detached with 0.1 mL trypsin (Trypsin-EDTA, EDTA—ethylenediaminetetraacetic acid, T4174, Sigma-Aldrich Co. Merck Group, Darmstadt, Germany) and each sample was suspended in 2 mL PBS. The fluorescence intensity of oxidized DCF was measured by integrating the signal for 5 s (λ_excitation_ = 488 nm, λ_emission_ = 523 nm, Fluorolog-2, Horiba Jobin-Yvon, Germany). The fluorescence was normalized to the number of cells from each sample (TC10™ Automated Cell Counter, Bio-Rad Laboratories Inc., Hercules, CA, USA). The oxidative stress level is normalized of that of the control cells grown w/o any compound. All measurements were performed in a minimum of triplicate. Statistical analysis of the data was performed using Student’s *t*-test on each pair of interest. Differences were considered statistically significant for which *p* < 0.05.

## 3. Results

### 3.1. Polyphenolic Extracts Characterization

The polyphenolic extracts were characterized using several spectrophotometric methods for the group quantification of polyphenols, flavonoids, anthocyanin pigments, as well as radical scavenger determination based on DPPH and ABTS assays (Table 1). The chemical profiling of prepared polyphenolic extracts was accomplished by reverse-phase HPLC-PDA analysis, in which were identified up to twelve compounds from 23 available standards.

The amount of extract and polyphenolic compounds recovered in each fraction obtained by conventional extraction decreased in each treatment stage. A slightly higher extraction yield was obtained for the conventional method (15.5% wt) than for the MW-assisted extraction (14.9% wt).

The total polyphenols content (TPC) was determined after removing interferents in order to obtain real TPC values. The TPC of fractions increased in each extraction stage ranging from 162.66 to 187.89 mg GAE/g extract. In the case of MM extracts prepared either by conventional or microwave-assisted extraction, TPC values were in the range of 196.32–203.98 mg GAE/g extract (30.51–30.32 mg GAE/g pomace).

The content of total reducing power substances (TRS) was higher for the extract prepared by conventional treatment (15.59 ± 0.41 mg AAE/g extract) than for the MW-extract (7.30 ± 0.31 mg AAE/g extract), proving that the MW extraction leads to a lower amount of reducing power substances in accordance with our previous results [49].

Higher amounts of ascorbic acid and total flavonoids (TFC) were obtained in the overall extract than for fractions. The TFC values for MM(Conv) and MM(MW) were in the range of 10.31–13.27 mg QE/g extract, a higher amount being found in the case of MW extract, while the anthocyanin pigments content (TAC) for overall extract and extract fractions were found to be similar.

Both extracts and fractions exhibited a good antioxidant activity with a concentration that inhibits 50% of DPPH free radical solution (IC50%) in the range of 0.94–1.12 mg/mL. However, IC50% values depend on the employed procedure, especially the concentration of DPPH solution. Thus, a better way to evaluate the radical scavenger activity is to express it in Trolox equivalents using two in vitro assays, DPPH and ABTS. For both MM(Conv) and MM(MW), similar antioxidant activity was observed being in the range of 246.58-289.96 mg TE/g extract for DPPH method (higher for MM(MW) extract) and 313.33–334.34 mg TE/g extract for ABTS assay (higher in the case of MM(Conv) extract), respectively.

**Figure 1:**
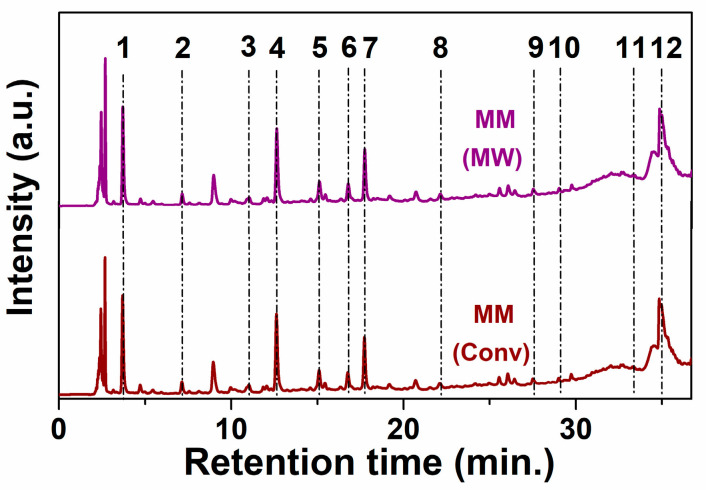
Chromatograms for MM(Conv) and MM(MW) extracts at 279 nm (1-gallic acid; 2-protocatechuic acid; 3-caftaric acid; 4-catechin hydrate; 5-vanilic acid; 6-syringic acid; 7-(−)epicatechin; 8-*p*-coumaric acid; 9-ellagic acid dihydrate; 10-rutin hydrate; 11-*trans*-resveratrol; 12-quercetin).

The chemical profiling of hydroethanolic polyphenolic extracts and extract fractions prepared by both conventional and microwave-assisted extraction was determined by reverse-phase HPLC-PDA (Figure 1 and Figure S1, respectively), using a method that we previously reported [49]. Four new standard substances in the calibration were added and the retention times, limit of detection and limit of quantification (Table S1) were presented in the Supplementary Material. From the available 23 standard substances, 12 were identified in the extracts (Table 2). As expected, one can observe that the polyphenols quantity decreases in each extraction stage, however an enhanced amount of quercetin and caftaric acid in the second and third stages were obtained.

### 3.2. Characterization of Pristine and Functionalized Silica-Type Carriers

**Figure 2:**
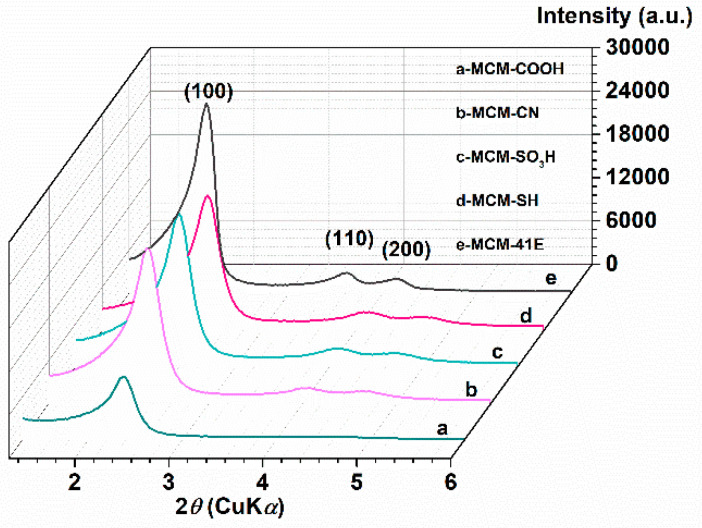
Small-angle X-ray diffraction (XRD) patterns for synthesized supports MCM-COOH(a); MCM-CN(b); MCM-SO_3_H(c); MCM-SH(d); MCM-41E(e).

As supports for loading the MM polyphenolic extract, pristine and functionalized MCM-41 materials were employed. The prepared mesoporous materials were characterized by different techniques. Thus, small-angle XRD patterns (Figure 2) proved that mesoporous MCM-41-type silica materials were obtained with an ordered hexagonal pore array, all showing an intense diffraction peak corresponding to the (100) Bragg reflection. For all materials, except MCM-COOH, the small-angle XRD analysis showed two additional Bragg reflections, (110) and (200), with lower intensity, specific for MCM-41-type materials. The silica surface functionalization with various organic moieties led to a shift towards lower *d_100_*-spacing values (Table 3).

**Figure 3:**
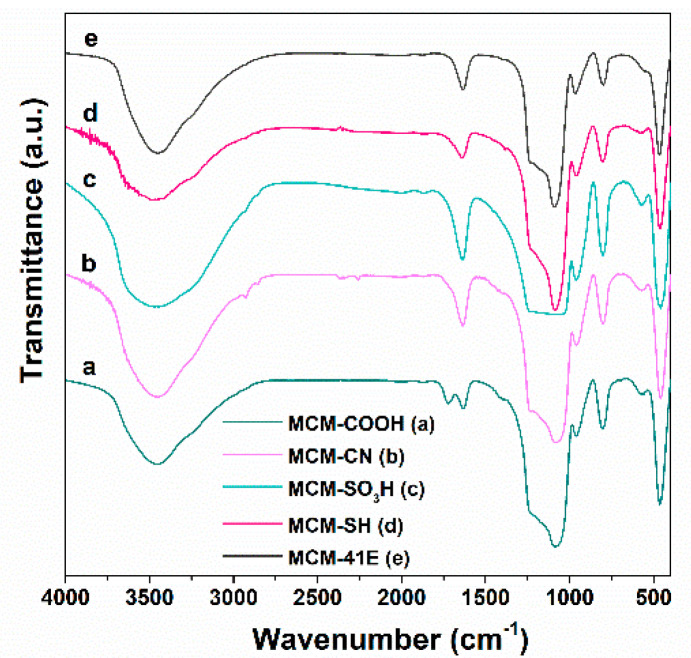
Fourier transform infrared (FTIR) spectra for synthesized supports MCM-COOH(a); MCM-CN(b); MCM-SO_3_H(c); MCM-SH(d); MCM-41E (e).

The FTIR spectroscopy evidenced the presence of organic groups linked on silica surface. In the FTIR spectra, all MCM-41-type supports (Figure 3) present the bands of silica matrix, asymmetrical and symmetrical stretching vibrations of Si-O-Si bonds at 1084 and 804 cm^−1^, respectively, stretching the vibration of silanol groups at 968 cm^−1^, and the deformation band of the Si–O bond at 468 cm^−1^, as well as the specific band of physiosorbed water molecules at 1640 cm^−1^. Also, in all FTIR spectra of functionalized silica materials (Figure 3a–e), asymmetrical and symmetrical methylene stretching vibrations in 2950–2800 cm^−1^ domain can be noticed. The MCM-COOH support (Figure 3a), obtained through a hydrolysis reaction from MCM-CN material, exhibited an intense band characteristic to the C=O bond of carboxylic group at 1726 cm^−1^ and no C–N stretching vibration at 2256 cm^−1^ can be seen as in the FTIR spectrum of the MCM-CN sample (Figure 3b), demonstrating the complete hydrolysis of -CN groups to -COOH moieties. In the case of MCM-SH material (Figure 3c), the vibration from 2550 cm^−1^ assigned to thiol groups is hard to notice because of its low intensity and small content of -SH groups (about 3.9% wt). In the FTIR spectrum of MCM-SO_3_H material (Figure 3d), the vibrations of sulfonic groups are superimposed with the very intense asymmetric stretching vibration of Si-O-Si, while in the spectrum of MCM-41E (Figure 3e) material, no specific bands of surfactant molecules used in the synthesis are observed demonstrating an efficient removal of the template agent by two-steps extraction.

**Figure 4:**
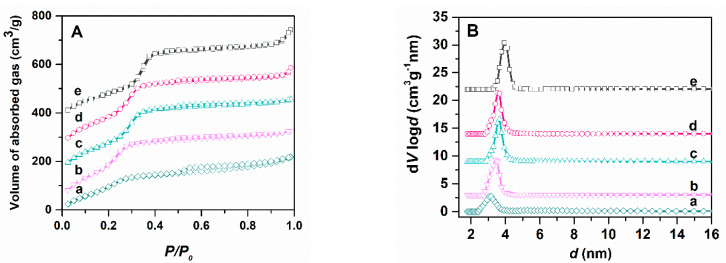
N_2_ adsorption-desorption isotherms of pristine and functionalized MCM-41 carriers (**A**) and their pore size distribution curves calculated with NLDFT (**B**): MCM-COOH (a); MCM-CN (b); MCM-SO_3_H (c); MCM-SH (d); MCM-41E (e)

To evaluate the porosity of silica-type supports, the most important feature that determines the quantity of phytocompounds that can be accommodated into the mesopores, pristine and functionalized MCM-41 samples were characterized by nitrogen adsorption–desorption isotherms (Figure 4A) that are type IV, with the H4 hysteresis loop at relative pressures higher than 0.5 due to the capillary condensation of nitrogen inside the mesopores, characteristic for mesoporous materials [55]. The pore size distribution curves (Figure 4B) were computed using the NLDFT model from the adsorption branch of the isotherms, being previously reported to better evaluate the pore diameter [52,56] (Figure 4A). The textural parameters of supports determined from N_2_ adsorption-desorption isotherms are listed in Table 3. After functionalization, the average pore size decreased compared to that of the commercial MCM-41 used for obtaining of functionalized mesoporous silica materials, more visible in the case of MCM-CN and MCM-COOH samples that have a higher amount of functional groups linked to the silica surface (between 7.5–8% wt) than MCM-SH or MCM-SO_3_H. The functionalized materials have lower specific surface area (585–845 m^2^/g) and total pore volume (0.74–0.43 cm^3^/g) than pristine silica (976 m^2^/g and 0.85 cm^3^/g, respectively).

The organic groups content of functionalized MCM-41-type supports was determined by thermogravimetric analysis (Figure S2) considering the weight loss up to 600 °C after subtracting the physically adsorbed water content at about 120 °C, which corresponded to the first endothermic event.

The morphology of MCM-41-type silica supports was evaluated by SEM. The synthesized MCM-41E support consists of ellipsoid-shaped particles with a diameter in the range of 170–310 nm and dimensions ratio of about 2 (Figure S3A). All functionalized MCM-41 materials were obtained from commercial MCM-41; thus, they have similar morphology presenting particles that form agglomerates with irregular shape and dimensions between 0.5–1.5 μm (Figure S3B–E). Because no significant differences in the FTIR spectra of MCM-SH and MCM-SO_3_H were observed, both functionalized supports were investigated by EDX analysis, which evidenced a slight difference in the sulfur content (Si/S = 30 for MCM-SH material and Si/S = 37 for MCM-SO_3_H sample) indicating that the oxidation reaction took place and some of mercaptopropyl moieties broke down during the oxidation process.

### 3.3. Characterization of Extract-Loaded Materials

The samples containing embedded extract were characterized by FTIR spectroscopy, which showed the presence of polyphenolic compounds into mesopores of the silica-type supports. The amount of phytocompounds adsorbed onto silica matrices was computed from thermal analysis (thermogravimetry/differential thermal analysis (TG-DTA)), while nitrogen adsorption-desorption isotherms were recorded to determine whether the mesopores of silica-type supports were filled completely with polyphenols. Also, the radical scavenger activity on solid samples was assessed to verify if the nanoconfinement of polyphenols into mesopores of the carrier improved the extract stability.

**Figure 5:**
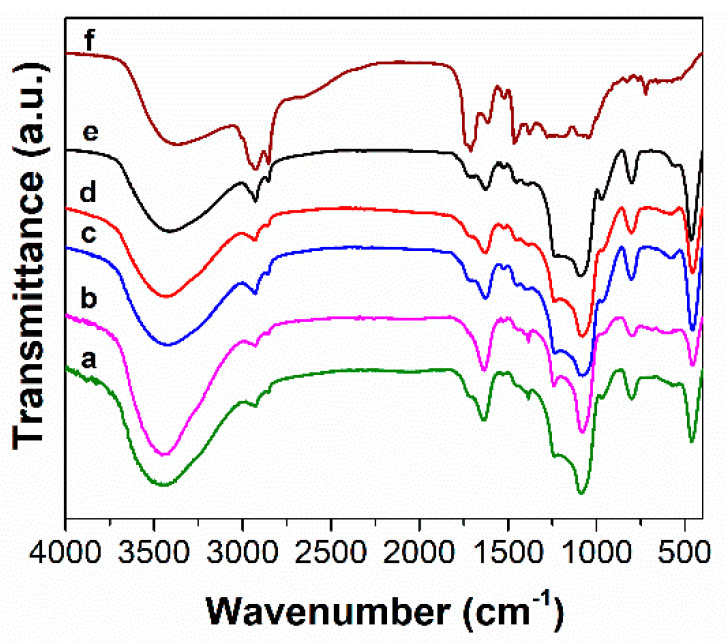
FTIR spectra of polyphenols-loading materials in comparison with the free extracts: MM@MCM-COOH(a); MM@MCM-CN(b); MM@MCM-SO_3_H(c); MM@MCM-SH(d); MM@MCM-41E(e); MM(Conv)(f).

In the FTIR spectra of materials containing polyphenolic extract (Figure 5a–e), one can notice the vibrations specific to the carrier and extract (Figure 5f). The bands assigned to the polyphenols are the stretching vibrations of C–H bonds in the 2830–3000 cm^−1^ domain and C-O bond (1724–1732 cm^−1^ for materials containing embedded extracts and 1712 cm^−1^ for polyphenolic extract), as well as bands specific to flavonoids, skeletal =C–O–C vibrations (1525–1527 cm^−1^ and 1522 cm^−1^ for polyphenols-loaded samples and free extract, respectively).

**Figure 6:**
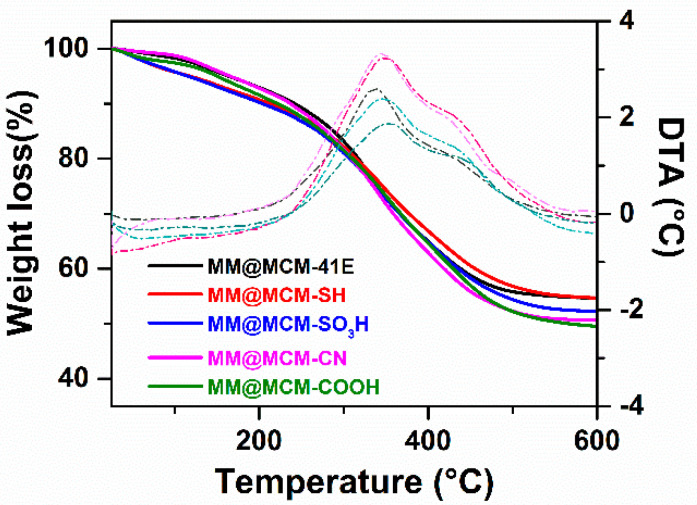
Thermogravimetry/differential thermal analyses (TG-DTA) of materials containing MM extract embedded in pristine and functionalized MCM-41 carriers.

Based on thermogravimetric analyses of extract-loaded samples (Figure 6), the content of polyphenolic compounds was determined deducting the weight loss of both adsorbed water and functional groups grafted on silica from the total mass loss up to 600 °C. The polyphenols content into the materials containing phytochemicals was in the range of 36–42% wt, the highest amount being obtained for the MM@MCM-41E sample, which contains the support with the highest pore volume (Table 3). The textural parameters of polyphenol-loaded materials are presented in Table 3. As can be seen, the total pore volume of the silica supports is almost filled with phytochemicals.

### 3.4. Radical Scavenger Activity of Extract-Loaded Materials

**Figure 7:**
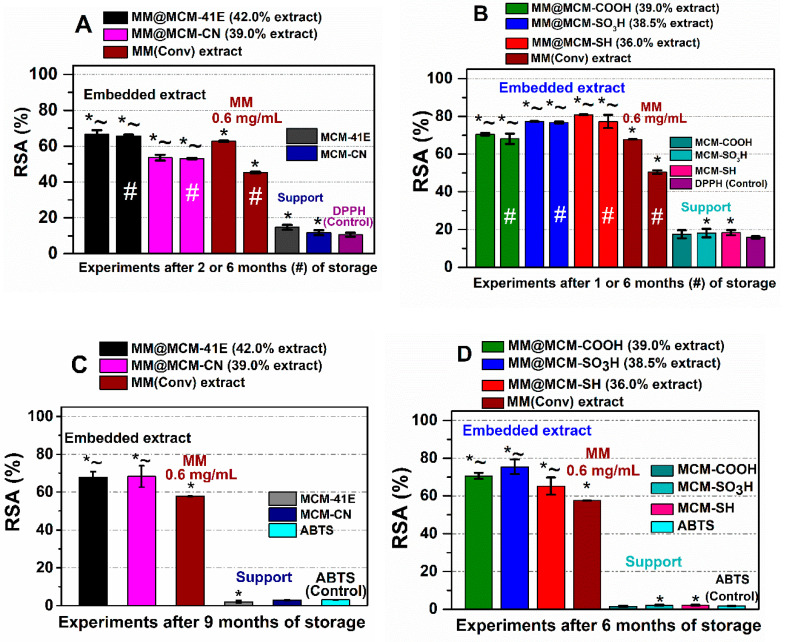
Radical scavenger activity on solid samples for embedded extracts in comparison with the corresponding support and free extract after 1–2 months or 6 months of storage (#), determined by DPPH assay (**A**,**B**) and after 6–9 months of storage using ABTS procedure (**C**,**D**). * *p* < 0.05 compared to control; ~ *p* < 0.05 compared to extract (Student *t*-test).

The radical scavenger activity of extract-loaded materials was determined after 24 h of incubation in DPPH free radical solution in dark conditions, in duplicate, and compared to that of the free extract and corresponding support in the same amount as in the materials containing the extract, using as control the degradation of the DPPH free radical solution. One can observe that the embedded extracts exhibited significantly higher (*p* < 0.05) radical scavenging activity after six months of storage than the free extract, whose capacity decreased in time (Figure 7).

For RSA determination using ABTS assay for solid samples, the stock solution of ABTS^•+^ was diluted with water to reach around 0.7 solution absorbance. It was considered that 6 h was enough for the extract-loaded materials to react with radical species from solution without any alteration of the mesoporous supports structure. The RSA values determined by ABTS assay are consistent with that determined through DPPH method, higher values were observed for MM(conv) extract embedded in all mesoporous supports after up to 9 months of storage in dark conditions, at 4 °C (Figure 7C,D).

### 3.5. Polyphenols Delivery Profiles from Pristine and Functionalized MCM-41 Supports

Firstly, the extract recovery experiment from MM@MCM-41E was performed in EtOH-water (4/1 *v*/*v*) mixture using a concentration of 0.5 mg/mL and then the recovered extract, MM(Conv)r, was analyzed by HPLC-PDA (Table 2). One can observe a higher amount of hydroxycinnamic acids and stilbenoids (by almost 25% and 40%, respectively), and a lower content of flavonoids and tannins (by 15% and almost 36%, respectively) than in the initial extract, which could be explained considering the transformation of natural compounds with high molecular weight into substances with lower molecular weight.

**Figure 8:**
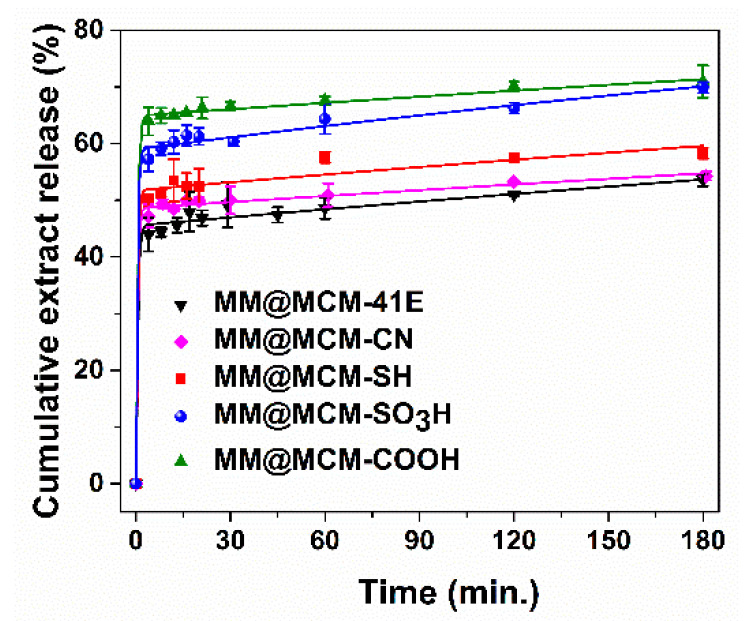
Polyphenols release profiles from MCM-41 silica-type supports fitted with three-parameter model.

Then, to investigate how the polyphenols are released from pristine and functionalized MCM-41 carriers in simulated body fluid depending on the surface properties of silica support and their possible biological effects, the extract delivery experiments were performed in phosphate buffer solution (PBS) pH 5.7. A partial recovery of polyphenols was achieved, the highest amount being recovered from MCM-COOH, 73.5 ± 1.3%, while the lowest from MCM-41E, 57.8 ± 0.6% (Figure 8).

### 3.6. Evaluation of Cellular Viability and Oxidative Stress Level

**Figure 9:**
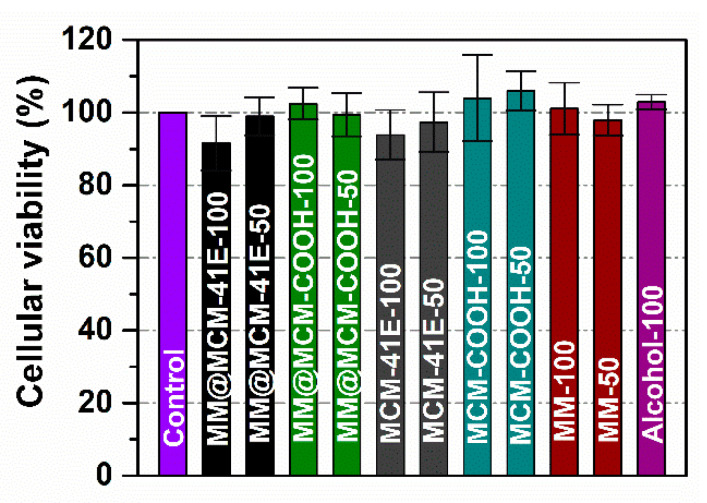
Cellular viability at 24 h of NIH3T3 cells (50 and 100 mean the sample concentration in μg/mL in growth medium). Data are presented as mean ± standard deviation (SD, *n* = 5). All samples did not significantly differ from Control (Student *t*-test).

The biocompatibility of MM polyphenolic extract free and loaded onto mesoporous silica materials, as well as of corresponding silica-type supports was assessed on NIH3T3 cells by MTS assay. Two concentrations of polyphenolic extract in growth medium, 50 μg/mL and 100 μg/mL, were tested. The tetrazolium salt-based reagent is converted by mitochondrial enzymes of viable cells into the soluble colored formazan product. Thus, the absorbance values of the formazan are directly proportional to the number of viable cells. The viability of cells treated with either MM polyphenolic extract or MM extract-loaded materials, as well as with silica supports is not significantly affected when compared to the control cells grown in the presence of any compound (Figure 9). At high concentration (100 μg/mL) of MM polyphenolic extracts loaded on MCM-41E supports, there is a slight decrease in the cell viability toward 90% from the Control. One may observe that this effect could be attributed to the silica support as a similar slight decrease of the cell viability is seen for the MCM-41E only. The alcohol from the extracts does not influence the cellular growth since the cells incubated with the same amount of alcohol as in the 100 μg/mL extract presents a viability like the Control.

**Figure 10:**
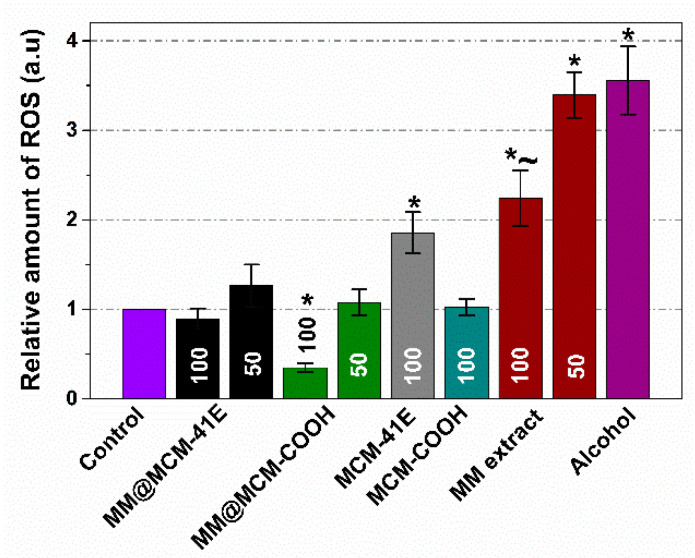
Intracellular reactive oxygen species (ROS) levels at 24 h on NIH3T3 (50 and 100 mean the sample concentration in μg/mL). Data are presented as mean ± SD (*n* = 3). * *p* < 0.05 compared to Control (cells grown w/o any compound); ~ *p* < 0.05 compared to alcohol (cells grown with alcohol in concentration corresponding to the highest concentration of ethanol from the extracts) (Student *t*-test).

One can observe a significant decrease of the ROS production for the MM@MCM-COOH sample (*p* < 0.05) for a concentration of 100 μg/mL when compared to that of the Control (Figure 10). The equivalent quantity of the MCM-COOH support does not significantly modify the level of ROS, thus the antioxidant effect of MM extract embedded on silica comes from the polyphenolic extract itself. The MCM-41E support (100 μg/mL) almost doubles the cytosolic ROS level with respect to Control. The scavenger activity of MM extract itself is (again) exerted here since 50 μg/mL polyphenolic extract embedded on the MCM-41E diminishes the oxidative effect of the support, while the concentration of 100 μg/mL extract is even annihilating it. The scavenger capacity of the extract was also present in the case of MM(Conv) extract only. For the concentration of 100 μg/mL MM(Conv) extract the ROS production was statistically significant diminished (*p* < 0.05) when compared to that of control cells treated with the same volume of ethanol as in MM(Conv) extract (Alcohol).

The production of reactive oxygen species (ROS) on NIH3T3 adherent cells was assessed using the 2′,7′-dichlorofluorescin diacetate-based assay. H_2_DCFDA is a membrane permeant non-fluorescent compound. Inside the cytosol, it is deacetylated by cellular esterase and later oxidized by ROS into a highly fluorescent compound. Thus, the fluorescent signal of DCF is directly proportional to the ROS production of the cytosol.

## 4. Discussion

In our study, grape pomace from Mamaia rosé winemaking process, a cultivar produced by the Research Station for Viticulture and Oenology Murfatlar (Black Sea region, Romania) was used as vegetal material. The extraction of polyphenolic compounds from Mamaia grape pomace was performed in 4/1 (*v*/*v*) ethanol/water mixture at reflux either by a conventional method or MW-assisted extraction. The recovered amount of polyphenols was significantly higher than that reported by Ruberto et al. for a grape pomace methanolic extract prepared at room temperature (3.4–5.8% wt) [57]. The conventional extraction proved to be more efficient based on the extract amount in comparison with the microwave-assisted method (15.5% vs. 14.9% wt).

The MM extracts prepared either by conventional or microwave-assisted extraction had TPC values in the range of 196.32–203.98 mg GAE/g extract (30.51–30.32 mg GAE/g pomace) that were higher than that reported for ethanol–water (4/1 *v*/*v*) extracts prepared by maceration at room temperature (60.3–131.7 mg GAE/g extract) [58], for Cabernet Sauvignon and Merlot pomace variety (61.4–64.8 mg GAE/g extract) [59], or for red wine grape pomace reported by Meini et al. (4.9 mg GAE/g pomace) [60], and lower than that obtained for an acidified ethanolic-water (2/5 *v*/*v*) extract prepared at 90 °C using MW power of 500 W/5 min (431.3 ± 3.6 mg GAE/ g extract) [61].

MW favored the extraction of flavonoids, but both prepared extracts had a high TPC being in the range of 1.60–2.06 mg QE/g pomace. The TFC value reported by Pintac et al. for extracts prepared from red wine grape pomace were significantly lower (0.24–0.65 mg QE/g pomace) than our values [59]. Being a rose wine grape pomace, MM hydroethanolic polyphenolic extracts obtained either by conventional extraction or MW-assisted method were not rich in anthocyanin pigments (12.85–13.03 mg CGE/g extract). Garcia-Becerra et al. reported significantly higher values for TAC (85.20 ± 0.80 mg CGE/g extract) for an ethanolic-water extract acidified with CH_3_COOH, which is most likely due to acidic medium extraction [61].

The prepared MM polyphenolic extracts exhibited a good radical scavenger activity in agreement with the TPC values, however the ABTS method led to higher RSA values. For MM(Conv) and MM(MW) similar RSA values were obtained, being in the range of 0.78–1.01 mmol TE/g extract for DPPH method (higher for MM(MW) extract) and 1.36–1.24 mmol TE/g extract (0.18–0.21 mmol TE/g pomace) for ABTS assay (higher for MM(Conv) extract). The RSA values are higher than that reported by Tournour et al. for an ethanolic-water (4/1 *v*/*v*) extract (0.52–1.09 mmol TE/g extract) using DPPH assay [58] or found by Meini et al. (0.03 mmol TE/g pomace) using ABTS method [60].

The chemical composition for MM hydroethanolic polyphenolic extracts was determined by reverse-phase HPLC-PDA analysis. Hence, gallic acid content varied from 324.26 to 328.61 mg/kg pomace, protocatechuic acid content ranged from 85.92 to 95.11 mg/kg pomace, caftaric acid amount was 120.86–123.19 mg/kg pomace, vanillic acid content was in the range of 162.91–164.92 mg/kg pomace and syringic acid content was 99.45–99.61 mg/kg pomace. Pintac et al. identified and quantified several polyphenolic compounds in Cabernet Sauvignon and Merlot ethanolic-water (4/1 *v/v*) extracts, such as gallic acid (0.57–0.61 mg/g extract), protocatechuic acid (0.010–0.045 mg/g extract), vanillic acid (0.125 ± 0.005 mg/g extract in Merlot extract), syringic acid (0.226–0.432 mg/g extract), all amounts being lower than that of our extracts [59]. A low gallic acid content of 0.146 mg/g extract and flavonoids, catechin and epicatechin, in traces, were reported by Peixoto et al. for methanol-water (4/1, *v*/*v*) extracts [62]. Also, a lower amount of gallic acid (86 ± 1.3 mg/kg pomace) than that obtained for our extracts was found in an ethanol-ethylene glycol extract [63], while Meini et al. reported a similar amount of gallic (300 mg/kg pomace) and 10 times higher amount of syringic acid (1300 mg/kg pomace) than in our extracts [60].

From the hydroxycinnamic group, only *p*-coumaric acid was identified and quantified in a higher amount (6.67–7.14 mg/kg pomace) than that reported by Pintac (0.003–0.010 mg/g extract) for ethanolic-water (4/1 *v*/*v*) extracts [59], but in lower quantity than that obtained by Meini et al. (20 mg/kg pomace) [60].

In our extracts, the polyphenols from flavonoids class were in the highest amounts, e.g., catechin hydrate (1511.47–1559.26 mg/kg pomace), (−) epicatechin (1048.04–1052.54 mg/kg pomace), rutin hydrate (105.66–114.50 mg/kg pomace), and quercetin (117.60–122.41 mg/kg pomace) being in higher quantities than that reported by Pintac et al. for ethanol-water (4/1 *v*/*v*) extracts (0.841 ± 0.008 mg/g extract catechin, 0.414–0.890 mg/g extract (−) epicatechin, 0.009–0.018 mg/g extract rutin and 0.661–0.759 mg/g extract quercetin) [59] or by Manconi et al. (86.4 ± 1.7 mg/kg pomace catechin, 97.6 ± 1.9 mg/kg pomace (−) epicatechin and 6.7 ± 0.2 mg/kg pomace quercetin) [63], but lower than obtained for a methanolic grape pomace extract by Ruberto et al. (0.54–2.77 mg/g extract quercetin) [57].

Ellagic acid hydrate from the tannins group and a stilbenoid, *trans*-resveratrol, were also identified in the MM(Conv) and MM(MW) extracts with a content in the range of 34.91–35.22 mg/kg pomace and 8.22–12.41 mg/kg pomace, respectively. Even though the amount of resveratrol found is very low in comparison with the other polyphenols, however, it is at least 5 times higher than that reported by Pintac et al. (0.009 ± 0.000 mg/g extract) [59].

A key factor in preserving the radical scavenger activity of polyphenolic extracts is to ensure their chemical and thermal stability, as well as the bioavailability. These could be accomplished by loading of polyphenolic compounds in various supports.

The hydroethanolic polyphenolic extracts prepared from Mamaia grape pomace either by conventional method or MW-assisted extraction had similar features. A better ABTS^•+^ scavenging activity was observed for MM(Conv), mainly due to a higher content of ascorbic acid, while for MM(MW) extract a higher TPC value and DPPH^•^ scavenging capacity were obtained. Due to a higher content of individual phenolic acids obtained for the MM(Conv) than in the case of MM(MW), except ellagic acid dihydrate, the MM(Conv) extract was chosen to be loaded in mesoporous MCM-41-type silica supports to improve its stability and delivery in order to be further applied in nutraceutical and cosmetic formulations.

For these proposed applications where the carriers should ensure a long-term preservation of phenolic compounds and their release from mesoporous matrices, and minimum toxic effects on cellular level, some prerequisite features for silica-type carriers were identified such as: silica surface functionalization for controlling the release of polyphenols, larger particles for avoiding the cellular uptake, and a simple and reliable obtaining route. For these reasons, either pristine MCM-41E rich in silanol groups to interact with phenolic compounds through hydrogen bonding, or functionalized mesoporous silica materials derived from commercial MCM-41, to form acid-base interactions with extract substances, were used. Hence, five mesoporous MCM-41-type materials, pristine and functionalized with mercaptopropyl, propyl sulfonic acid, cyanoethyl and propionic acid moieties, were employed for loading MM(Conv) extract.

All MCM-41-type mesoporous supports had an ordered hexagonal pore array, proved by small-angle XRD, and can accommodate a large amount of phenolic compounds (36–42% wt) due to their high pore volume (0.43–0.74 cm^3^/g), the main feature responsible for this. Concerning the morphology of MCM-41 silica supports, the MCM-41E material presents ellipsoid-shaped particles with a diameter of 170–310 nm and a dimensions ratio of approximately 2, while all functionalized MCM-41 materials prepared from commercial MCM-41 consist of particles in the range of 0.5–1.5 μm (Supplementary Material, Figure S3).

The stability over the time of MM(Conv) hydroethanolic polyphenolic extract when loaded in pristine and functionalized MCM-41 supports was evaluated by radical scavenging activity determination (DDPH and ABTS assays). The RSA values of embedded extracts for ABTS assay were higher when compared with the DPPH method because of different solvent, pH and nature of free radicals, which influence the scavenging capacity of polyphenolic compounds. However, the RSA values higher for ABTS method than for DPPH assay obtained for extract-loaded materials are consistent with the behavior of the free MM(Conv) extract. The extract loaded in all MCM-41-type supports preserved significant better the radical scavenger capacity, being almost constant after several months of storage in dark conditions, at 4 °C than the free extract, whose antioxidant activity decreased over the time (Figure 7). These results demonstrate an improved stability of extract when confined in silica mesopores, considering that the supports did not exhibit an important effect on both DPPH^•^ and ABTS^•+^ scavenging capacity. The mesoporous supports presented very low RSA values (higher up to 2.5% and 0.63% for DPPH^•^ and ABTS^•+^ controls, respectively, except MCM-41E). The effect of MCM-41E support on radicals scavenging capacity (7.6% higher than DPPH control) can be explained by its higher adsorption capacity of organic species due to larger pore volume than that of functionalized silica carriers. Unlike the other materials containing embedded extract, a lower RSA was observed for the MM@MCM-CN sample than that for the free extract after two months of storage (Figure 7A), probably because of the strong base nature of CN groups associated with most likely strong acid–base interactions between phenolic acids and cyanoethyl groups grafted on MCM-CN support. However, even though this support was less efficient in terms of interactions with phytocompounds, after six months of storage, the DPPH^•^ scavenging capacity of MM@MCM-CN sample remained almost constant in comparison with that of the free extract, whose antioxidant activity decreased significantly. Also, the ABTS^•+^ scavenging capacity of MM@MCM-CN sample after nine months of storage was higher that of the free extract.

The polyphenol release profiles were assessed in PBS pH 5.7. In order to evaluate the correlation between polyphenolic compounds delivery process and the nature of functional organic groups linked on silica-type support, the experimental data were fitted using a three-parameter model (Figure 8) [64], which considers an equilibrium between adsorbed and desorbed organic molecules and the diffusion process, all having a first order kinetics that facilitate the transport of phytochemicals inside the support meso-channels and then into the release medium. The free molar enthalpy, Δ*G*, is a parameter that characterized the adsorption (*k_on_*)/desorption (*k_off_*) equilibrium, being proportional to the amount of phytochemicals released in the first stage of the release process (burst effect), when the molecules that interact weakly with the silica surface are able to diffuse rapidly into the release medium, and the rate constant for diffusion, *k*_d_, is proportional to the polyphenols delivery rate [65].

Regarding Δ*G* values (Table 4), the lowest polyphenols amount delivered in the first stage of the release process is observed from pristine silica, followed by supports functionalized with organic groups with base nature (MM@MCM-CN and MM@MCM-SH). The highest burst effect was noticed for carriers with acidic groups, especially for the MM@MCM-COOH sample for which the highest phenolic compounds recovery yield was reached.

**Figure 11:**
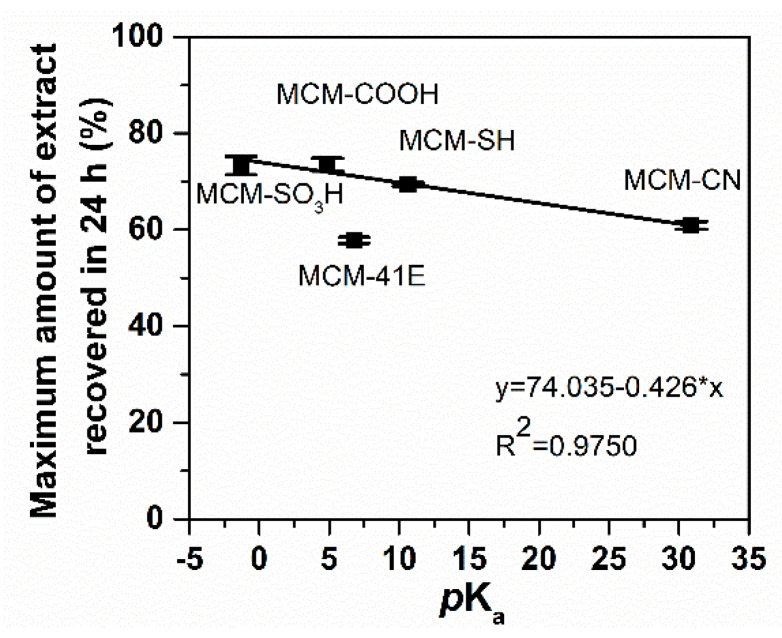
Dependence of maximum amount of phenolic compounds recovered on pKa values of organic groups grafted on silica support.

However, a tendency for re-adsorption of polyphenols into the pores of silica-type carrier was noticed for some samples, *k*_on_ > *k*_off_, this process being more evident for pristine and functionalized silica with basic organic groups, cyanopropyl and mercaptopropyl, which established strong acid–base interactions with phenolic acids from extract. The polyphenols delivery is favored as the rate constant of diffusion, *k_d_*, is three orders of magnitude higher than the adsorption and desorption constant rates. The increase of acidity of organic groups linked on silica pore walls surface favored the polyphenols delivery due to weaker acid–base interactions between support and phenolic acids that are in a high amount in MM(Conv) extract, the pKa value of organic groups being inversely proportional with the phytochemicals amount delivered in PBS pH 5.7 (Figure 11).

Concerning the biocompatibility of mesoporous silica materials, cytotoxicity studies showed that particles size and shape may affect their cellular uptake depending on cell type and culturing media, but silica surface properties influence more clearly the biocompatibility, which is related to silanol groups. Through functionalization of silica surface in which silanol groups are involved in bonding organic groups, the concentration of silanol groups decreased [66]. For instance, He et al. reported a higher toxicity for spherical silica particles with a size of 190 or 420 nm than that of microparticles with 1220 nm diameter over a broad range of concentration (10–480 μg/mL) on either African green monkey kidney COS-7 cells or the human breast-cancer MDA-MB-468 cell line. Larger silica particles reduced cellular uptake, which could be responsible for lowering the cytotoxicity of microparticles [67].

The cytotoxicity of MM polyphenolic extract free and embedded onto mesoporous MCM-41 silica matrices, as well as of corresponding supports was evaluated on NIH3T3 cell line (MTS assay). One can notice that the cellular viability was not significantly affected when compared to the control cells (cells grown w/o any compound) for both cells grown in the presence of free or embedded extracts (Figure 9). The MTS assay was performed after the free/embedded extracts were removed from the culture and the cells washed. Thus, only the cells that “lived together” with the free/encapsulated extracts, continued to have NADH reserves and functional enzymes at the level of mitochondria were able to produce the formazan-colored compound. In conclusion and in accordance to the ISO 9001 standard the materials containing embedded MM extract and the hydroalcoholic polyphenols extract did not present in vitro toxic effects on NIH3T3 cell line for 24 h exposure time and concentrations up to 100 μg/mL.

In the last few decades, polyphenols have been studied intensively due to their antioxidant properties. Polyphenols interact with proteins and metallic ions inhibiting their signaling pathway. Also, they act as scavengers for cellular radicals and, therefore, they lower the oxidative stress [68]. Unfortunately, polyphenols have a poor intestinal absorption and their stability in biological medium is low. They are easily metabolized inside the organism and hence, the beneficial cellular activity is partially lost [69].

The intracellular ROS production level when NIH3T3 cells were treated with MM polyphenolic extract free and loaded on mesoporous supports (Figure 10) correlates with the polyphenol release profiles from MCM-41 silica-type supports (Figure 8): the carrier with acidic groups (MM@MCM-COOH) led to the highest polyphenols release profile, for this sample the antioxidant effect of MM extract loaded on MCM-COOH is the most prominent. Moreover the ROS production levels measured for MCM-41E and MCM-COOH supports are in accordance with the RSA of these materials (Figure 7): for instance, the ROS production level is higher for MCM-41E, which presents a RSA with 4.0% more than control unlike the ROS production level of MCM-COOH support that exhibits a RSA with 1.0% higher than control (no significant difference from control).

An enhanced oxidative stress was observed for control cells treated with ethanol (approximately 3.5 times higher than Control). The cells treated with the MM polyphenolic extract showed a lower ROS production level than alcohol control, especially in the case of the high concentration (100 μg/mL). However, both concentrations of MM extract induced a much higher oxidative stress than in control cells grown *w/o* any compound. Recently, it has been reported that at 37 °C there is a significant decrease of stability and structural modification of the polyphenols, which could explain their partial loss of antioxidant properties [70]. This may explain the increase in oxidative stress level in the case of cells incubated with extract only with respect to those incubated with the extract embedded on supports: the extract confined in silica mesopores are more stable. This also may explain the apparent lack of correlation of oxidative stress measurements with the assessment of radical scavenger activity performed at room temperature, which showed that at several months of storage the MM extract has almost the same RSA as the MM extract embedded on MCM-COOH and MCM-41E supports (Figure 7). One may consider that the increased stability of MM extract observed when it was confined in silica mesopores (at 6 months storage the RSA of embedded extracts was similar to the level of 1 or 2 months) may also be exerted when working at 37 °C. Anyway in conditions of good stability of the extract when embedded on silica supports, the scavenger activity of the MM extract itself was always exerted (less prominent at 50 μg/mL extract, but importantly at 100 μg/mL extract embedded) when comparing to the support.

Due to the potential use of polyphenols in various fields, there is a constant requirement in enhancing the cellular uptake of polyphenols, but also an improvement of their structural stability. The polyphenols-loaded MCM-41-type silica, especially the MM@MCM-COOH sample, developed within our study showed an improved stability over time and also a good in vitro antioxidant effect on NIH3T3 cells, being alternative candidates to the classical delivery of polyphenols within biological systems.

## 5. Conclusions

The hydroalcoholic polyphenolic extracts from Mamaia cultivar, prepared by conventional or microwave-assisted extraction, showed a high amount of total polyphenols (196.32–203.98 mg GAE/g extract), flavonoids (10.31–13.27 mg QE/g extract), and anthocyanins (12.85–13.03 mg CGE/g extract) being correlated with a good radical scavenging activity (246.58–334.34 mg TE/g extract).

To delay the decrease of radical scavenger activity in time, the polyphenolic extract prepared by conventional extraction was embedded for the first time in mesoporous silica functionalized with organic groups. The proposed materials containing phytochemicals showed better RSA in comparison with the free extract after several months of storage, being good candidates for cosmetic or nutraceutical formulations.

The acidic properties of functional groups grafted on silica supports influenced the amount of polyphenolic compounds released in PBS pH 5.7, the increase of functional groups pKa value lowering the amount of recovered phytochemicals.

The cellular viability experiments on NIH3T3 fibroblast cells proved no significant toxicity for polyphenol-loaded materials. The intracellular reactive oxygen species assay correlated well with the release in PBS of the polyphenolic compounds: the lowest cytosolic ROS production was observed in the case of MM@MCM-COOH material, which showed the highest amount of phytochemicals released in PBS pH 5.7. Moreover, the polyphenols-loaded MCM-41-type silica, especially MM@MCM-COOH sample, showed an improved stability over time and a better in vitro antioxidant effect than the polyphenolic extract alone.

## Figures and Tables

**Table 1:** Total polyphenols content (as gallic acid equivalents, GAE), total reducing substances (TRS, as ascorbic acid equivalents, AAE), total flavonoids content (as quercetin equivalents, QE), total anthocyanin pigments (TAC, as cyanidin-glucoside equivalents, CGE) and radical scavenging activity by both 2,2-diphenyl-1-picrylhydrazyl (DPPH) and 2,2′-azino-bis(3-ethylbenzothiazoline-6-sulphonic acid (ABTS) methods (as Trolox equivalents, TE) for MM extracts.

Extract	Extract (% wt)	TPC (mg GAE/ g)	TRS (mg AAE/ g)	TFC (mg QE/ g)	TAC (mg CGE/g)	IC50% (mg/ mL)	RSA _DPPH_ (mg TE/ g)	RSA _ABTS_ (mg TE/ g)
MM FI	9.0	162.66 ± 1.59	16.86 ± 0.51	13.24 ± 1.37	7.51 ± 0.68	1.12	242.77 ± 5.93	208.02 ± 6.47
MM FII	4.4	167.74 ± 3.26	16.97 ± 1.33	9.31 ± 0.28	7.55 ± 0.44	1.02	265.74 ± 11.90	212.34 ± 5.03
MM FIII	2.7	187.89 ± 3.64	16.84 ± 0.42	8.17 ± 0.00	8.20 ± 0.13	0.97	280.19± 15.52	254.31 ± 9.06
MM (Conv)	15.5	196.32 ± 1.85	15.59± 0.41	10.31 ± 0.86	12.85 ± 0.23	1.10	246.58 ± 6.04	334.34 ± 14.45
MM (MW)	14.9	203.98 ± 1.15	7.30 ± 0.31	13.27 ± 0.04	13.03 ± 0.70	0.94	289.96 ± 10.47	313.33 ± 8.46

All values are expressed per gram of extract unless stated otherwise.

**Table 2:** Polyphenolic compounds identification and quantification by reverse-phase HPLC-PDA.

Standard Substances	Concentration in Extract (mg/g)					
	MM- Fr I	MM- Fr II	MM- Fr III	MM (Conv)	MM (MW)	MM (Conv)r
gallic acid	2.130 ± 0.003	1.821 ± 0.000	1.561 ± 0.001	2.118 ± 0.002	2.090 ± 0.000	2.420 ± 0.011
protocatechuic acid	0.532 ± 0.000	0.483 ± 0.001	0.464 ± 0.002	0.613 ± 0.002	0.577 ± 0.002	0.640 ± 0.004
caftaric acid	0.552 ± 0.000	0.857 ± 0.006	1.085 ± 0.006	0.794 ± 0.000	0.779 ± 0.004	0.318 ± 0.000
catechin hydrate	11.344 ± 0.016	9.964 ± 0.002	9.909 ± 0.032	10.050 ± 0.004	9.742 ± 0.000	8.571 ± 0.263
vanillic acid	1.238 ± 0.003	1.015 ± 0.001	0.818 ± 0.001	1.050 ± 0.003	1.063 ± 0.002	1.547 ± 0.006
syringic acid	0.654 ± 0.002	0.496 ± 0.002	0.416 ± 0.003	0.641 ± 0.000	0.642 ± 0.001	0.722 ± 0.012
(−) epicatechin	7.536 ± 0.017	6.672 ± 0.000	6.525 ± 0.002	6.784 ± 0.002	6.755 ± 0.012	5.560 ± 0.009
*p*-coumaric acid	nd	nd	nd	0.046 ± 0.001	0.043 ± 0.000	0.057 ± 0.002
ellagic acid dihydrate	0.237 ± 0.001	0.253 ± 0.003	0.212 ± 0.000	0.225 ± 0.000	0.227 ± 0.001	0.143 ± 0.000
rutin hydrate	0.739 ± 0.000	0.702 ± 0.001	0.555 ± 0.001	0.681 ± 0.000	0.738 ± 0.001	0.814 ± 0.000
*trans*-resveratrol	nd	nd	nd	0.053 ± 0.000	0.080 ± 0.000	0.075 ± 0.000
quercetin	0.805 ± 0.001	0.936 ± 0.001	1.166 ± 0.003	0.789 ± 0.001	0.758 ± 0.001	0.619 ± 0.009

nd—not detected. All values are given in mg compound/g of extract.

**Table 3:** Textural parameters of supports and their corresponding extract-loaded materials.

Support Type	Support				MM@support			
	n_SiO2_/n_FG_	*d*_DFT_ (nm)	*S*_BET_ (m^2^/g)	*V*_p_ (cm^3^/g)	Extract (% wt)	*d*_DFT_ (nm)	*S*_BET_ (m^2^/g)	*V*_p_ (cm^3^/g)
MCM-41E	-	3.93	781	0.69	42.0	3.66	97	0.11
MCM-SH	25	3.54	843	0.74	36.0	3.42	231	0.16
MCM-SO_3_H	50	3.66	798	0.59	38.5	3.54	141	0.10
MCM-CN	11	3.18	845	0.55	39.0	-	75	0.06
MCM-COOH	14	3.18	585	0.43	39.0	-	10	0.04

FG-functional groups; d_DFT_-average pore size values computed from non-local density functional theory (NLDFT) method, S_BET_- specific surface area determined by the Brunauer Emmett Teller (BET) method, V_p_-total pore volume, measured at *p*/*p*_0_ = 0.99. Commercial MCM-41 (*d*_DFT_ = 3.66 nm; *S*_BET_ = 976 m^2^/g; *V*_p_ = 0.85 cm^3^/g).

**Table 4:** Kinetic parameters for polyphenols release from MCM-41-type supports.

Materials Containing Embedded Extract	Three-Parameter Fitting Equation					Maximum Amount of Extract Recovered (%)
	Δ*G* (10^21^J)	*k*_d_ (min^−1^)	*k*_off_ (10^3^min^−1^)	*k*_on_ (10^3^min^−1^)	*R* ^2^	
MM@MCM-41E	−0.76	1.940	0.899	1.073	0.9936	57.8 ± 0.6
MM@MCM-SH	0.31	2.256	0.985	0.917	0.9934	69.4 ± 0.4
MM@MCM-SO_3_H	1.57	2.256	1.756	1.218	0.9975	73.3 ± 1.9
MM@MCM-CN	−0.07	2.256	0.721	0.732	0.9981	70.0 ± 1.4
MM@MCM-COOH	2.63	1.938	1.129	0.610	0.9993	73.5 ± 1.3

## Supplementary Materials

The following are available online at https://www.mdpi.com/2076-3921/9/8/696/s1: Figure S1: HPLC-PDA chromatogram for MM extracts obtained in each extraction stage at 279 nm; Figure S2: Thermogravimetric analysis of pristine and functionalized MCM-41 carriers; Figure S3: scanning electron microscopy (SEM) micrograph for MCM-41E (A), MCM-CN (B), MCM-COOH (C), MCM-SH (D) and MCM-SO3H (E), Table S1: HPLC-PDA characterization of a standard solution mixture.
