# Supplementary material for: Effect of Nanoconfinement of Polyphenolic Extract from Grape Pomace into Functionalized Mesoporous Silica on Its Biocompatibility and Radical Scavenging Activity
Source: Antioxidants (Basel). 2020 Aug 3;9(8):696. doi: 10.3390/antiox9080696 (PMC7465047; doi:10.3390/antiox9080696)
Supplement: Supplementary file 1 [file antioxidants-09-00696-s001.pdf]

1. HPLC-PDA analysis of standard solution mixture and MM extract fractions

In the Mamaia extract fractions the same ten compounds were identified from which the first fraction mostly presented the highest amount of polyphenolic, hydroxycinnamic acids and flavonoids except caftaric acid and quercetin content, which were in the highest amount in the third fraction that sustained the highest radical scavenger activity of third fraction.

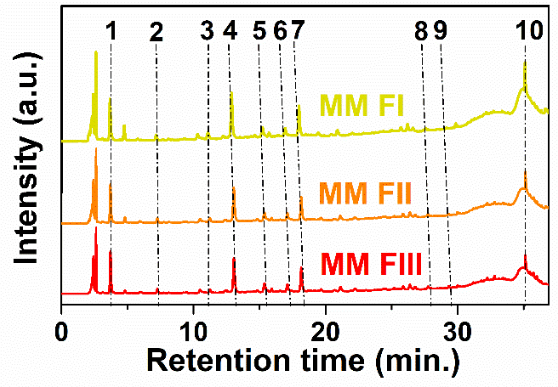

Figure S1. HPLC-PDA chromatogram for MM extracts obtained in each extraction stage at 279 nm (1-gallic acid; 2-protocatechuic acid; 3-caftaric acid; 4-catechin hydrate; 5-vanilic acid; 6-syringic acid; 7-(-)epicatechin; 8-ellagic acid dihydrate; 9-rutin hydrate; 10-quercetin).

HPLC-PDA characterization of a standard solution mixture of twenty-three polyphenolic compounds is presented in Table S1, being mentioned their retention times, maximum absorption wavelength, limit of detection and quantification, as well as the linearity domain.

Table S1. HPLC-PDA characterization of a standard solution mixture.

Compound	Retention Time (min.)	Calibration curve	R^2	λ (nm)	LOD (mg/L)	LOQ (mg/L)	Linearity Domain (mg/L)
Gallic acid	3.702	y=8371.14*x-1207.10	0.9996	271	0.108	0.540	0.54-108.00
Protocatechuic acid	7.158	y=9007.30*x-1186.03	0.9998	279	0.099	0.494	0.49-98.80
Caftaric acid	10.920	y=3741.63*x-1004.52	0.9990	326	0.100	0.500	0.50-100.00
Catechin hydrate	12.645	y=1704.55*x-1484.97	0.9987	279	0.520	1.040	0.52-104.00
Chlorogenic acid	13.393	y=7634.01*x-1686.39	0.9992	326	0.099	0.495	0.49-99.00
Vanillic acid	15.122	y=8457.44*x-1216.69	0.9995	292	0.099	0.494	0.49-98.70
Caffeic acid	15.383	y=13188.1*x-1501.42	0.9994	323	0.099	0.495	0.49-99.00
Syringic acid	16.803	y=6929.03*x-667.27	0.9991	271	0.107	0.537	0.54-107.30
(-) Epicatechin	17.763	y=1585.49*x-273.15	0.9993	279	0.468	0.935	0.94-93.50
Delphinidin chloride	21.212	y=13373.7*x-5554.22	0.9984	529	0.118	0.590	0.59-118.00
trans p-Coumaric acid	22.166	y=18220.6*x-1642.10	0.9972	309	0.102	0.510	0.51-102.00
Trans-ferulic acid	25.455	y=13317.51*x-3135.95	0.9997	323	0.104	0.520	0.52-104.00
Ellagic acid dihydrate	25.621	y=5128.27*x-577.18	0.9995	367	0.102	0.510	0.51-71.12
Cyanidin chloride	25.668	y=13401.6*x-2555.96	0.9990	524	0.084	0.420	0.42-84.00
Rutin hydrate	26.599	y=3813.02*x-838.902	0.9992	355	0.499	0.998	0.99-99.80
Chicoric acid	29.175	y=10560.62*x-1939.03	0.9996	330	0.102	0.510	0.51-101.60
Pelargonidin chloride	29.642	y=8921.84*x-2511.85	0.9993	512	0.100	0.500	0.50-100.00
Malvidin chloride	30.899	y=7243.86*x-2363.29	0.9983	535	0.092	0.460	0.46-92.00

Myricetin	31.874	y=9150.32*x-1464.36	0.9995	373	0.095	0.475	0.48-95.00
Rosmarinic acid	32.359	y=7282.31*x-633.293	0.9988	330	0.099	0.495	0.49-99.00
trans-Resveratrol	33.317	y=17601.1*x-2585.25	0.9994	307	0.100	0.500	0.50-100.00
Quercetin	34.865	y=9898.83*x-723.173	0.9997	371	0.099	0.495	0.49-99.00
Kaempferol	35.875	y=10549.7*x-1296.00	0.9990	367	0.097	0.485	0.48-97.00

LOD-limit of detection; LOQ-limit of quantification.

2. Thermogravimetric analysis

The thermal analyses of pristine and functionalized mesoporous silica are presented in Figure S2. The content of organic groups linked on silica pore walls was determined considering the weight loss up to 600°C and neglecting the weight loss associated with the first endothermic event, attributed to the physiosorbed humidity up to 120°C.

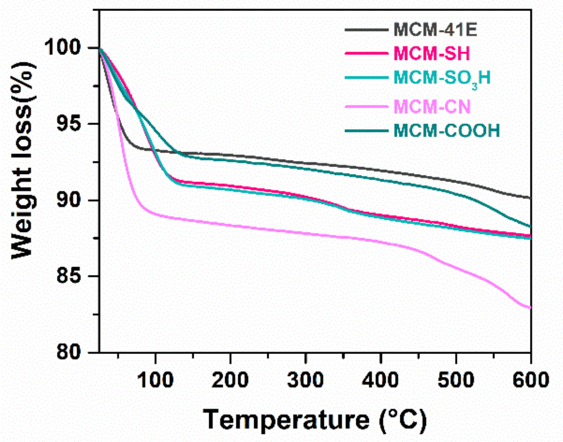

Figure S2. Thermogravimetric analysis for functionalized MCM-41 carriers.

3. Determination of mesoporous supports morphology by scanning electron microscopy (SEM)

The morphology of MCM-41-type supports was evaluated by SEM analysis. The synthesized MCM-41E carrier consists of ellipsoidal-shaped particles with a diameter in the range of 170-310 nm and a dimensions ratio of about 2 (Figure S3-A) and no significant changes in morphology were noticed between the functionalized samples, obtained from commercial MCM-41, consisting of particles that form agglomerates with irregular shapes and dimensions ranging from 0.5 to 1.5 μm (Figure S3 B-E).

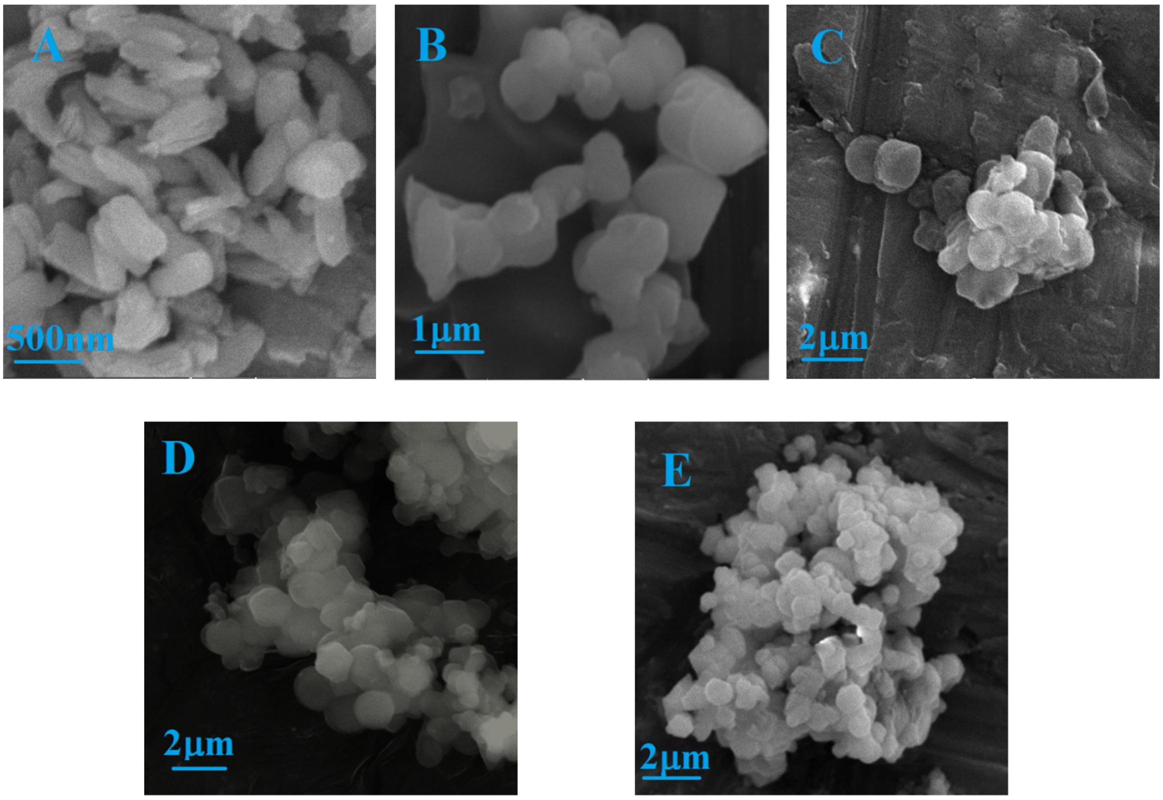

Figure S3. SEM micrograph for MCM-41E (A), MCM-CN (B), MCM-COOH (C), MCM-SH (D) and MCM-SO_3H (E).
